# The prognostic value of an autophagy-related lncRNA signature in hepatocellular carcinoma

**DOI:** 10.1186/s12859-021-04123-6

**Published:** 2021-04-28

**Authors:** Shiming Yang, Yaping Zhou, Xiangxin Zhang, Lu Wang, Jianfeng Fu, Xiaotong Zhao, Liu Yang

**Affiliations:** 1 Shandong Second Provincial General Hospital, Shandong, China; 2 Shihezi University School of Medicine, Shihezi, China; 3 The First Affiliated Hospital of Shihezi University School of Medicine, Shihezi, China; 4 Clinical Laboratory Diagnosis Center, General Hospital of Xinjiang Military Region, Xinjiang, China; 5 Department of Medical Laboratory Science, Xinjiang Bayingoleng Mongolian Autonomous Prefecture People’s Hospital, Xinjiang, China

**Keywords:** Hepatocellular carcinoma, Autophagy-related lncRNAs, The Risk prediction model

## Abstract

**Background:**

lncRNA may be involved in the occurrence, metastasis, and chemical reaction of hepatocellular carcinoma (HCC) through various pathways associated with autophagy. Therefore, it is urgent to reveal more autophagy-related lncRNAs, explore these lncRNAs’ clinical significance, and find new targeted treatment strategies.

**Methods:**

The corresponding data of HCC patients and autophagy genes were obtained from the TCGA database, and the human autophagy database respectively. Based on the co-expression and Cox regression analysis to construct prognostic prediction signature.

**Results:**

Finally, a signature containing seven autophagy-related lncRNAs (PRRT3-AS1, RP11-479G22.8, RP11-73M18.8, LINC01138, CTD-2510F5.4, CTC-297N7.9, RP11-324I22.4) was constructed. Based on the risk score of signature, Overall survival (OS) curves show that the OS of high-risk patients is significantly lower than that of low-risk patients (*P* = 2.292e−10), and the prognostic prediction accuracy of risk score (AUC = 0.786) is significantly higher than that of ALBI (0.532), child_pugh (0.573), AFP (0.5751), and AJCC_stage (0.631). Moreover, multivariate Cox analysis and Nomogram of risk score are indicated that the 1-year and 3-year survival rates of patients are obviously accuracy by the combined analysis of the risk score, child_pugh, age, M_stage, and Grade (The AUC of 1- and 3-years are 0.87, and 0.855). Remarkably, the 7 autophagy-related lncRNAs may participate in Spliceosome, Cell cycle, RNA transport, DNA replication, and mRNA surveillance pathway and be related to the biological process of RNA splicing and mRNA splicing.

**Conclusion:**

In conclusion, the 7 autophagy-related lncRNAs might be promising prognostic and therapeutic targets for HCC.

**Supplementary Information:**

The online version contains supplementary material available at 10.1186/s12859-021-04123-6.

## Introduction

Hepatocellular carcinoma (HCC), as the most common type of liver cancer [1], with high cancer-related mortality and poor prognosis worldwide [2], makes a threat to public health. Alcohol, aflatoxin, and hepatitis can increase HCC risk, and genetic and epigenetic changes can promote malignancy [3, 4]. At present, surgical resection remains the most common treatment option for patients with HCC. Because of tumor metastasis and relapse [5], the prognosis is dissatisfying. Most patients with advanced HCC often have low 5-year survival [6]. Currently, molecular alterations of HCC have been reported in some studies [7], and those molecular mechanisms can be explored further in the diagnosis and treatment of HCC [8–10]. However, these efforts have not made a significant improvement in patient survival. Therefore, it is necessary to identify more potential biomarkers that may be related to HCC carcinogenesis.

As a highly selective quality control, mechanism autophagy is the key to maintaining homeostasis in physiological and pathological conditions [11], such as adapting to metabolic stress, removing dangerous cargo, and renovating during differentiation and development, genomic prevention damage [12]. However, more and more evidence indicated that hat autophagy could also help tumor occurrence, maintenance, and development [13]. In pancreatic cancer [14], autophagy is a metabolic requirement for cancer cells' immune evasion, allowing tumors to achieve optimal proliferation in vitro and vivo. Besides, autophagy is necessary for tumor cell migration and metastasis because its inhibition will block cell migrates and metastasis [15]. In HCC, autophagy can promote tumor cells' metastasis by upregulating the expression of MCT1 and the activation of the Wnt/β-catenin signaling pathway [16]. The epithelial-mesenchymal can also be activated by autophagy to promote cancer cell invasion [17]. Thus some researchers have tried to find new targeted treatment strategies for HCC by studying autophagy pathways.

Long non-coding RNAs (lncRNAs) are a class of RNA transcripts that consist of more than 200 nucleotides in length and exhibit limited protein-coding capacity [18]. However, With in-depth exploration, lncRNA has been found to perform essential functions in various biological processes such as post-transcriptional regulation, transcriptional regulation, and chromatin modification [19]. Some studies have also reported that lncRNAs regulate many aspects of cancer progression and affect different malignant behaviors, such as cancer cell proliferation, apoptosis, and metastasis [20, 21]. Remarkably, owing to the complexity and diversity, lncRNA's abnormal expression will promote the occurrence of a variety of tumors, such as cervical carcer [22], esophageal squamous cell carcinoma [23], and lung adenocarcinoma [24]. Autophagy has been considered to play a dual and contradictory role in carcinogenesis, and this exact mechanisms that result in autophagy in cancer still need to be further explored [25]. lncRNA participates in the occurrence, invasion, metastasis, prognosis, and chemoresistance of HCC by regulating various pathways related to autophagy [26–28]. However, these studies only concentrated on single or a few lncRNAs for HCC. Although some studies have explored the impact of autophagy-related lncRNAs on the prognosis of HCC patients and constructed predictive signatures, the accuracy of each signature is different and the lncRNAs included are not all the same [29–32]. Therefore, are there other autophagy-related lncRNAs that affect the prognosis of HCC patients? Compared with traditional features, how accurate is the prognosis prediction signature based on these lncRNAs? Is the signature based on HCC patients also applicable to intrahepatic cholangiocarcinoma (ICC) patients? What are the important biological functions of these lncRNAs and what signal transduction pathways do they participate in? These are the questions that our research needs to explore.

## Materials and methods

### Data obtaining and processing

The entire sequencing profile data of patients with liver hepatocellular carcinoma (HCC) were obtained from The Cancer Genome Atlas (TCGA, https://cancergenome.nih.gov/) database. According to the gene annotations from the GENCODE project (https://www.gencodegenes.org/) [33], the lncRNA and protein-coding gene profile data were further classified. Besides, the patient's corresponding clinical information was downloaded from the TCGA database, such as survival time, survival status, age, gender, tumor grade, and TNM stage. Moreover, other special clinical features of HCC including prothrombin time (PT), bilirubin, albumin, AFP and Child–Pugh grade were obtained as well. Based on the previously study of Johnson et al. [34], the Albumin–Bilirubin (ALBI) score was also considered into this study to further assess the performance advantage of our constructed signature. ALBI score (As) is calculated as = (log10 bilirubin * 0.66) + (− 0.085 * albumin), where bilirubin is in umol/L and albumin in g/L, ALBI grade 1 ≤ − 2.6, ALBI grade 2: − 2.6 < As ≤ − 1.39, and ALBI grade 3: As > − 1.39. And then, the HCC patients with incomplete clinical information and survival time that less than 30 days were removed. The sequencing data and clinical information of patients with intrahepatic cholangiocarcinoma (ICC) were obtained and processed in the same way. Since all of these data involved in this study were publicly available, the ethics committee has no specific ethical approval.

### Screening of autophagy-related lncRNAs

A list of the autophagy-related genes was downloaded from the Human Autophagy Database (HADb, http://www.autophagy.lu/). The autophagy gene expression profile was extracted from the fore mentioned protein-coding gene profile data. To identify the potential lncRNA related to autophagy-related genes, we performed a Pearson correlation analysis in the lncRNA and autophagy-related gene expression profile by “limma” package [35]. The thresholds were set as follows: ∣R| > 0.4 and *P* < 0.001 were considered a strong correlation.

### Identify prognosis-related autophagy lncRNAs and calculate the risk score

To confirm the potential prognostic value of autophagy-related lncRNAs, based on the “survival” package [36], univariate Cox regression analysis and product-limit method (Kaplan–Meier method) were used to assess the association between autophagy-related lncRNA expression and survival data. Those autophagy-related lncRNAs are significantly related to survival (Both Kaplan–Meier method and univariate Cox regression satisfy *P* value < 0.01) were obtained as prognosis-related lncRNAs. Those prognosis-autophagy-related lncRNAs were then used into multivariate Cox regression analysis to obtain regression coefficients (β) with the lowest Akaike information criterion (AIC) values and then establish a risk score. The risk score calculation based as follows = βlncRNA1 × ExpressionlncRNA1 + βlncRNA2 × ExpressionlncRNA2 + … + βlncRNA1n × ExpressionlncRNAn. According to the median risk score, the patients of HCC and ICC were classified into high-risk and low-risk groups respectively to further assess whether this signature of risk score is suitable for two types of liver cancer. The “pheatmap” [37] package was utilized to draw the survival status of HCC patients and the heatmap of lncRNA expression based on risk scores grade.

### Analysis of risk score model

According to the "survival" package, the univariate and multivariate Cox regression analysis were utilized to evaluate whether the risk score of the autophagy-related lncRNAs can be an independent indicator for the prognosis of HCC. And then, based on “survivalROC” [38] package, the receiver operating characteristic (ROC) curve and area under the ROC curve (AUC value) were performed to evaluate diagnostic efficacy of risk score compared with the traditional clinical features (child_pugh, ALBI, APF, TNM_stage, stage, grade, PT, bilirubin, and albumin) in HCC and ICC patients. In order to further evaluate the performance advantage in our signature, the " survivalROC " package was used to calculate the AUC values of 1-, 2-, 3-, 4- and 5- year both in our signature and the previously published lncRNAs signature in HCC 06-09, and plot the results through the " ggplot2 " package [39].

### Evaluation and construction of Nomogram

Based on the result of multivariate Cox regression analysis, the “survival” and “rms” [40] packages were utilized to construct a Nomogram which can predict survival probability of HCC patients by the combination of risk score and clinical data. And then, the Bootstrap self-sampling method was repeated 1000 times to evaluate whether the consistency of the predicted results and the actual results by the internal validation method. The difference between the predicted results of the nomogram and the actual results were drew in the calibration curve. Finally, calculated the time dependent ROC curves and the AUC values of this Nomogram.

### Functional analysis

To further explore the Gene Ontology (GO) [41] and the Kyoto Encyclopedia of Genes and Genomes (KEGG) pathways [42], these prognostic autophagy-related lncRNAs may participate. We performed Pearson correlation analysis in the final prognostic autophagy-related lncRNA and protein-coding gene profile data. The thresholds were set as follows: ∣R| > 0.4 and *P* < 0.001. Then we analyzed those co-expressed genes with prognostic autophagy-related lncRNAs through the “clusterProfiler" [43] package to speculate the pathways and biological processes that lncRNA may participate in.

## Result

### Acquisition of autophagy-related lncRNAs

A total of 147 patients with complete clinical information, 14,748 lncRNAs, and 19,767 mRNA were screened from the TCGA-HCC. In the TCGA-ICC dataset, there were 36 patients have complete survival data. Moreover, 232 autophagy genes were obtained from HADb database, among which 213 genes were expressed in TCGA-HCC. Finally, a total of 557 autophagy-related lncRNAs were identified from TCGA-HCC according to the Pearson correlation analysis with the screening criteria of ∣R| > 0.4 and *P* < 0.001 (Additional file 1: Table S1).

### The prognostic autophagy-related lncRNA

**Fig. 1:**
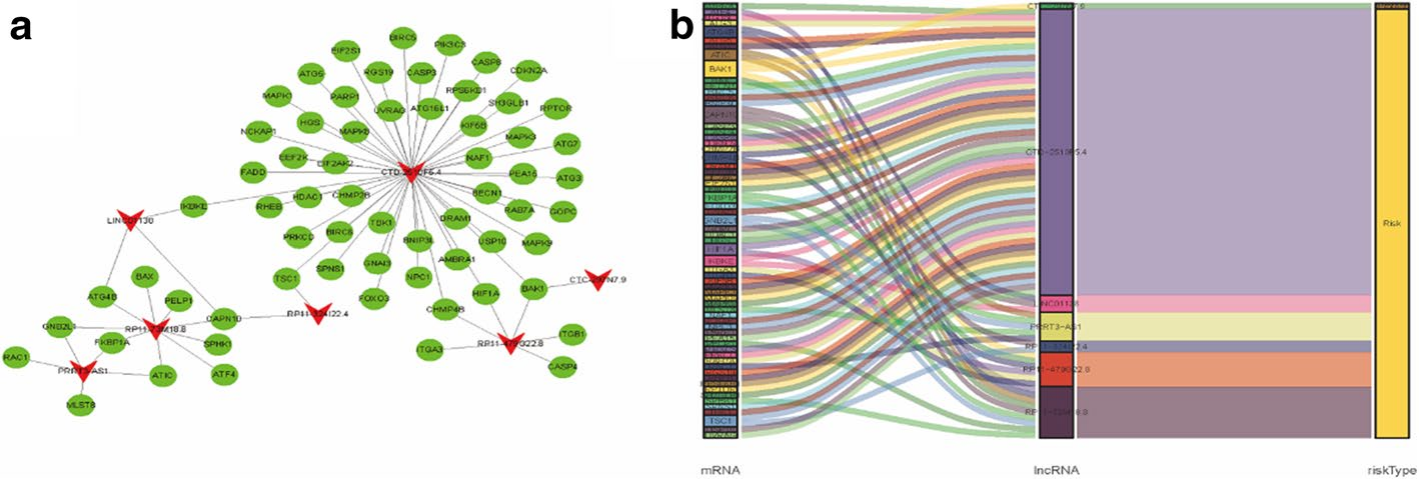
The Co-expression network and Sankey diagram of prognostic autophagy-related lncRNAs. **a** The Co-expression network between prognostic autophagy-related lncRNAs and autophagy-related genes in Hepatocellular carcinoma. The green circle nodes represent prognostic autophagy-related lncRNAs, and the red V nodes represent autophagy-related genes. The Co-expression network was visualized using Cytoscape 3.7.2 software. **b** Sankey’s diagram showed the relationship between prognostic autophagy-related lncRNAs, autophagy genes, and risk types

**Fig. 2:**
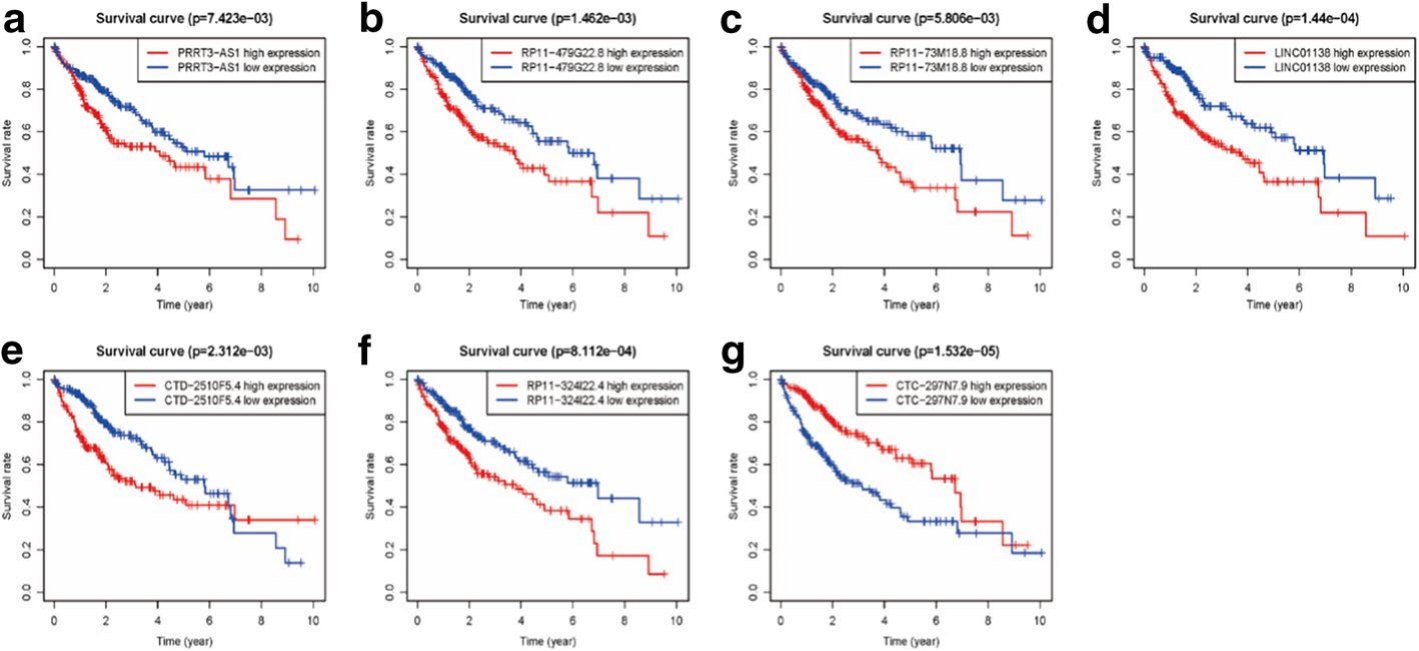
The KM survival curves of 7 prognostic autophagy-related lncRNAs. **a** PRRT3-AS1, **b** RP11-479G22.8, **c** RP11-73M18.8, **d** LINC01138, **e** CTD-2510F5.4, and **f** RP11-324I22.4 were harmful prognostic factors, and the **g** CTC-297N7.9 was a favorable prognostic factor

**Fig. 3:**
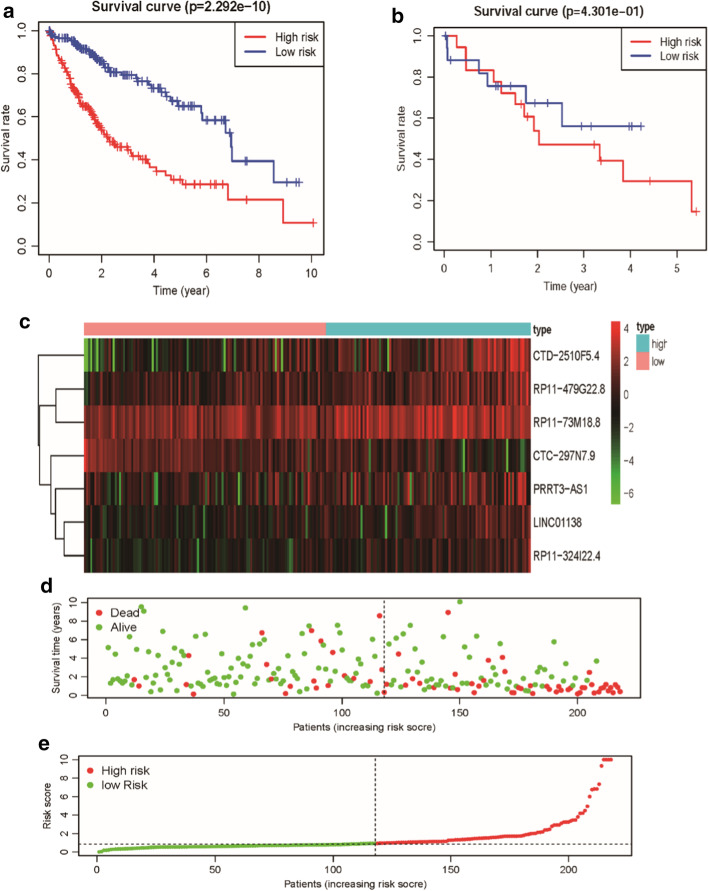
The performance verification of constructedd signature. **a** The KM survival curve indicates that the OS of HCC patients in the high-risk group is significantly lower than that of low-risk patients (*P* = 2.292e−10). **b** The KM survival curve indicates that there is no difference in OS of ICC patients between in the high-risk group and low-risk group (*P* = 4.301e−01). **c** The Heatmap of 7 autophagy-related lncRNAs' expression in the low- and high-risk groups. **d** The scatterplot of the risk scores and the survival status/survival time. Red represents dead; green represents alive. **e** The ranked risk score of all autophagy-related lncRNAs. Red represents a high risk; green represents a low risk

According to univariate Cox regression analysis and product-limit method (*P* value < 0.01), 39 autophagy-related lncRNAs which related to prognostic have been selected (Additional file 2: Table S2). Subsequently, seven autophagy-related lncRNAs that had a co-expression relationship with 65 autophagy genes were identified as robust independent prognostic factors after Multivariate Cox regression (Fig. 1). Among them, PRRT3-AS1 (HR 1.1028, 95% CI 1.0284–1.1827), RP11-479G22.8 (HR 1.0936, 95% CI 1.0261–1.1656), RP11-73M18.8 (HR 1.0635, 95% CI 1.0070–1.1233), LINC01138 (HR 1.2752, 95% CI 1.0110–1.6085), CTD-2510F5.4 (HR 1.1355, 95% CI 1.0607–1.2157), and RP11-324I22.4 (HR 1.2956, 95% CI 1.0181–1.6488) were unfavorable prognostic factors, and the CTC-297N7.9 (HR 0.577, 95% CI 0.3363–0.9900) was beneficial prognostic factor (Fig. 2 and Table 1). The risk score of each HCC patient = (0.0979*PRRT3-AS1) + (0.0896*RP11-479G22.8) + (0.0616*RP11-73M18.8) + (0.2431*LINC01138) + (0.1271*CTD-2510F5.4) + (− 0.5400*CTC-297N7.9) + (0.2590*RP11-324I22.4). Based on the risk score, the overall survival (OS) curves indicates that the OS of the high-risk group for HCC patients was significantly shorter than the low-risk group (*P* = 2.292e−10) (Fig. 3a). The five years survival rate of the high-risk group (HR 0.286, 95% CI 0.199–0.411) is less than half of the low-risk group (HR 0.694, 95% CI 0.547–0.77). Similarly, the distributions of survival status also indicated that the survival rate and time of HCC high-risk group are significantly lower than the low-risk group (Fig. 3d, e), and the expression of 6 unfavorable prognostic factors (PRRT3-AS1, RP11-479G22.8, RP11-73M18.8, LINC01138, CTD-2510F5.4, and RP11-324I22.4) is increased with the risk score increases; on the contrary, the expression of beneficial prognostic factor (CTC-297N7.9) is decreased (Fig. 3c). However, the signature constructed by the sequencing data of HCC patients is deed not suitable for ICC patients, because the OS curve indicates that there is no difference in OS between ICC patients in the high- and low-risk groups (*P* = 4.301e−01) (Fig. 3b).

**Table 1:** The HR, 95% CI of HR, and *P* value of the 7 autophagy-related lncRNA based on the multivariate Cox regression analysis

Name	HR	95% CI of HR	*P* value
PRRT3-AS1	1.1028	1.0284–1.1827	0.0061
RP11-479G22.8	1.0936	1.0261–1.1656	0.0059
RP11-73M18.8	1.0635	1.0070–1.1233	0.0272
LINC01138	1.2752	1.0110–1.6085	0.0401
CTD-2510F5.4	1.1355	1.0607–1.2157	0.0003
CTC-297N7.9	0.577	0.3363–0.9900	0.0459
RP11-324I22.4	1.2956	1.0181–1.6488	0.0352

*HR* hazard ratio

### Clinical value of the prognostic autophagy-related lncRNA

**Fig. 4:**
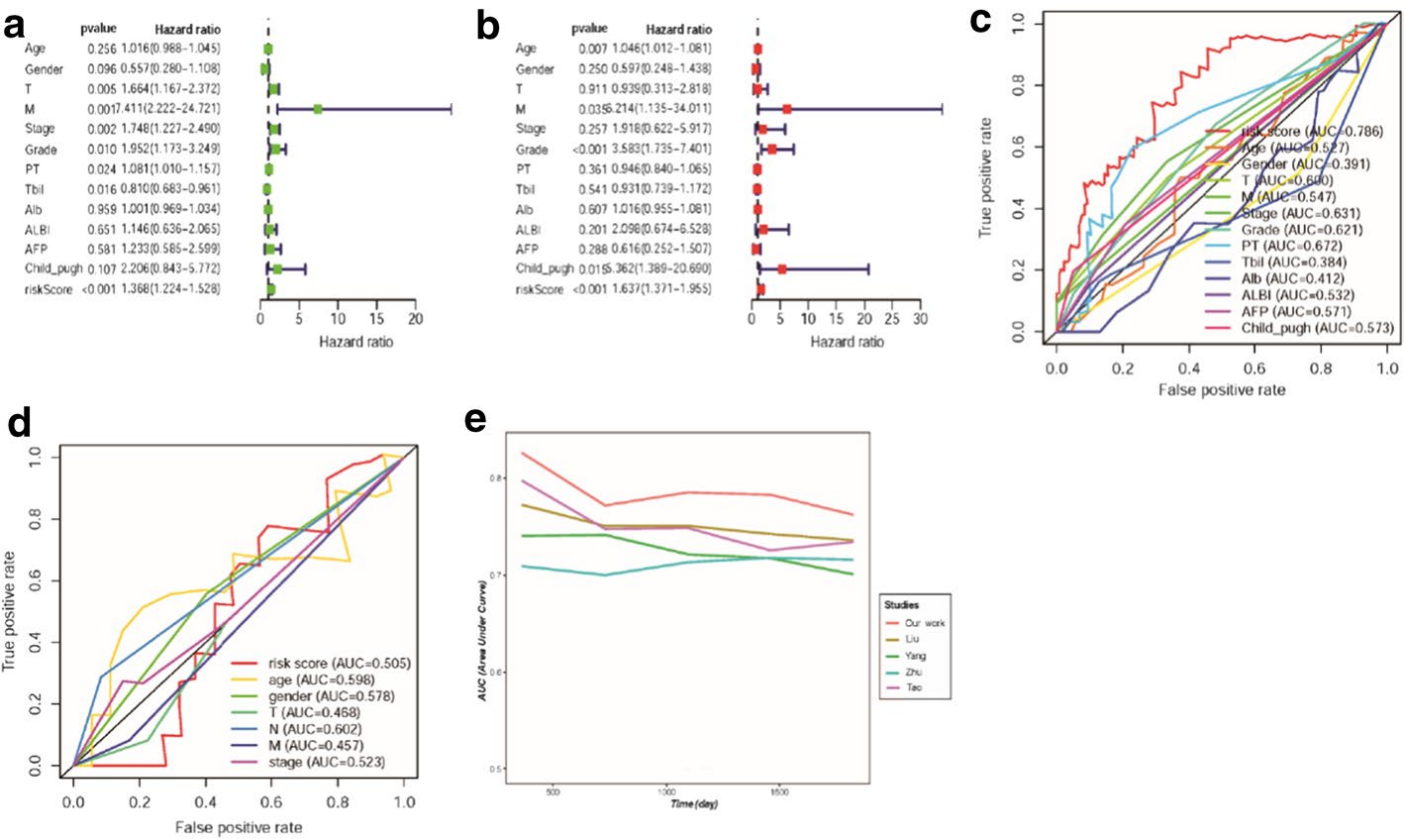
The performance evaluation of constructed signature. **a** The green forest plot indicates the univariate Cox analysis in the clinical features, risk score and OS of HCC patients. The risk score, T_stage, M_stage, Stage, Grade, prothrombin time (PT), and toal bilirubin (Tbil) are associated with the prognosis of HCC patients (*P* = 2.292e−10). **b** The red forest plot indicates the multivariate Cox analysis in the clinical features, risk score and OS of HCC patients. The risk score, M_stage, Age, and child_pugh are related to the prognosis of HCC patients (*P* = 4.301e−01). **c** The 3-year time-dependent ROC curve of risk score in the HCC patients (AUC = 0.786). **d** The 3-year time-dependent ROC curve of risk score in the ICC patients (AUC = 0.505). **e** The performance evaluation between multiple predictive signatures. The signature of each study is represented by the family name of the corresponding author

To evaluate whether the seven autophagy-related lncRNAs could be used as the independent prognosis biomarkers of patients in HCC, Univariate cox regression analysis, and Multivariate cox regression analysis were utilized to evaluate the relationship between the clinical data and risk score. Univariate Cox regression indicated thatT (*P* = 0.005, 95% CI 1.167–2.372), M (*P* = 0.001, 95% CI 2.222–24.721), Stage (*P* = 0.002, 95% CI 1.227–2.490), Grade (*P* = 0.01, 95% CI 1.173–3.249), PT (*P* = 0.024, 95% CI 1.010–1.157), Tbil (*P* = 0.016, 95% CI 0.683–0.961), risk score (*P* < 0.001, 95% CI 1.224–1.528) were related to the prognosis (Fig. 4a and Table 2). However, after implying multivariate analysis, only the risk score (*P* < 0.001, 95% CI 1.371–1.955), Age (*P* = 0.007, 95% CI 1.012–1.081), M (*P* = 0.035, 95% CI 1.135–34.011), Grade (*P* < 0.001, 95% CI 1.735–7.401), child_pugh (*P* = 0.015, 95% CI 1.389–20.690) were associated with prognosis (Fig. 4b and Table 2). Next, the receiver operating characteristic (ROC) curves were utilized to evaluate the risk score's predictive performance. In HCC group, the area under the ROC (AUC) curve of risk score for 3-years is 0.786 (Fig. 4c), however, the AUC of ALBI, AFP, and Child–Pugh are only 0.532, 0.571, and 0.573 respectively. Similarly, the AUC of risk score for 3-years is only 0.505 In ICC group as well (Fig. 4d). In the predict performance comparison of multiple signatures, the predict performance of our constructed signature is superior to the previously published 4 lncRNA-related signature in HCC (Fig. 4e, Table 3).

**Table 2:** Clinical characteristics and risk scores based on Univariate and multiple Cox regression analysis

Type	B	SE	z	HR	95% CI of HR	*P* value
Univariate Cox regression analysis						
Age	0.016	0.014	1.135	1.016	0.988–1.045	0.256
Gender	0.585	0.351	1.667	0.557	0.280–1.108	0.096
T	0.509	0.181	2.815	1.664	1.167–2.372	**0.005**
M	2.003	0.615	3.259	7.411	2.222–24.721	**0.001**
Stage	0.559	0.180	3.095	1.748	1.227–2.490	**0.002**
Grade	0.669	0.260	2.573	1.952	1.173–3.249	**0.010**
PT	0.078	0.035	2.253	1.081	1.010–1.157	**0.024**
Tbil	0.210	0.087	2.413	0.810	0.683–0.961	**0.016**
Alb	0.001	0.016	0.052	1.001	0.969–1.034	0.959
ALBI	0.136	0.300	0.453	1.146	0.636–2.065	0.651
AFP	0.210	0.380	0.552	1.233	0.585–2.599	0.581
Child_pugh	0.791	0.491	1.613	2.206	0.843–5.772	0.107
Risk score	0.313	0.057	5.520	1.368	1.224–1.528	**< 0.0001**
Multiple Cox regression analysis						
Age	0.045	0.017	2.708	1.046	1.012–1.081	**0.007**
Gender	0.515	0.448	1.150	0.597	0.248–1.438	0.250
T	0.063	0.561	0.112	0.939	0.313–2.818	0.911
M	1.827	0.867	2.106	6.214	1.135–34.011	**0.035**
Stage	0.651	0.575	1.133	1.918	0.622–5.917	0.257
Grade	1.276	0.370	3.448	3.583	1.735–7.401	**0.001**
PT	0.055	0.061	0.914	0.946	0.840–1.065	0.361
Tbil	0.072	0.118	0.612	0.931	0.739–1.172	0.541
Alb	0.016	0.032	0.514	1.016	0.955–1.081	0.607
ALBI	0.741	0.579	1.280	2.098	0.674–6.528	0.201
AFP	0.485	0.457	1.062	0.616	0.252–1.507	0.288
Child_pugh	1.679	0.689	2.437	5.362	1.389–20.690	**0.015**
Risk score	0.493	0.091	5.438	1.637	1.371–1.955	**< 0.0001**

The bold means these clinical features are statistically significant in the Univariate- or Multivariate-cox regression analysis

**Table 3:** Performance evaluation among 5 lncRNAs-related prognostic prediction models for HCC patients

Signature	Risk score	The AUC value				
		1-year	2-year	3-year	4-year	5-year
7-lncRNAs (Our study)	Risk score = (0.0979 × PRRT3-AS1) + (0.0896 × RP11-479G22.8) + (0.0616 × RP11-73M18.8) + (0.2431 × LINC01138) + (0.1271 × CTD-2510F5.4) + (-0.5400 × CTC-297N7.9) + (0.2590 × RP11-324I22.4)	0.826	0.772	0.785	0.783	0.762
7-lncRNAs (Liu's study)	Risk score = (0.3563 × AC005229.4) + (0.2698 × AL365203.2) + (0.3230 × AL117336.3) + (0.2081 × AC099850.3) + (0.2852 × ELFN1-AS1) + (0.2428 × LUCAT1) + (0.5044 × AL031985.3)	0.773	0.751	0.751	0.743	0.736
5-lncRNAs (Yang's study)	Risk score = (CYTOR × 0.17456) + (LINC01063 × 0.30093) + (MKLN1-AS × 0.27462) + (PLBS1-AS1 × 0.17218) + (TMMC1-AS1 × 0.28974)	0.740	0.742	0.722	0.718	0.701
4-lncRNAs (Zhu's study)	Risk score = (0.125 × AC099850.3) + (0.109 × LUCAT1) + (0.055 × ZFPM2‐AS1) + (0.106 × AC009005.1)	0.709	0.700	0.714	0.718	0.716
4-lncRNAs (Tao's study)	Risk score = (0.2567 × RP11-322E11.5) + (0.1307 × RP11-150O12.3) + (− 0.2320 × AC093609.1) + (− 0.1857 × CTC-297N7.9)	0.798	0.748	0.749	0.726	0.734

This table covers the linear calculation formula and the AUC value within 1- to 5- years of each signature

### Evaluation of the nomogram

**Fig. 5:**
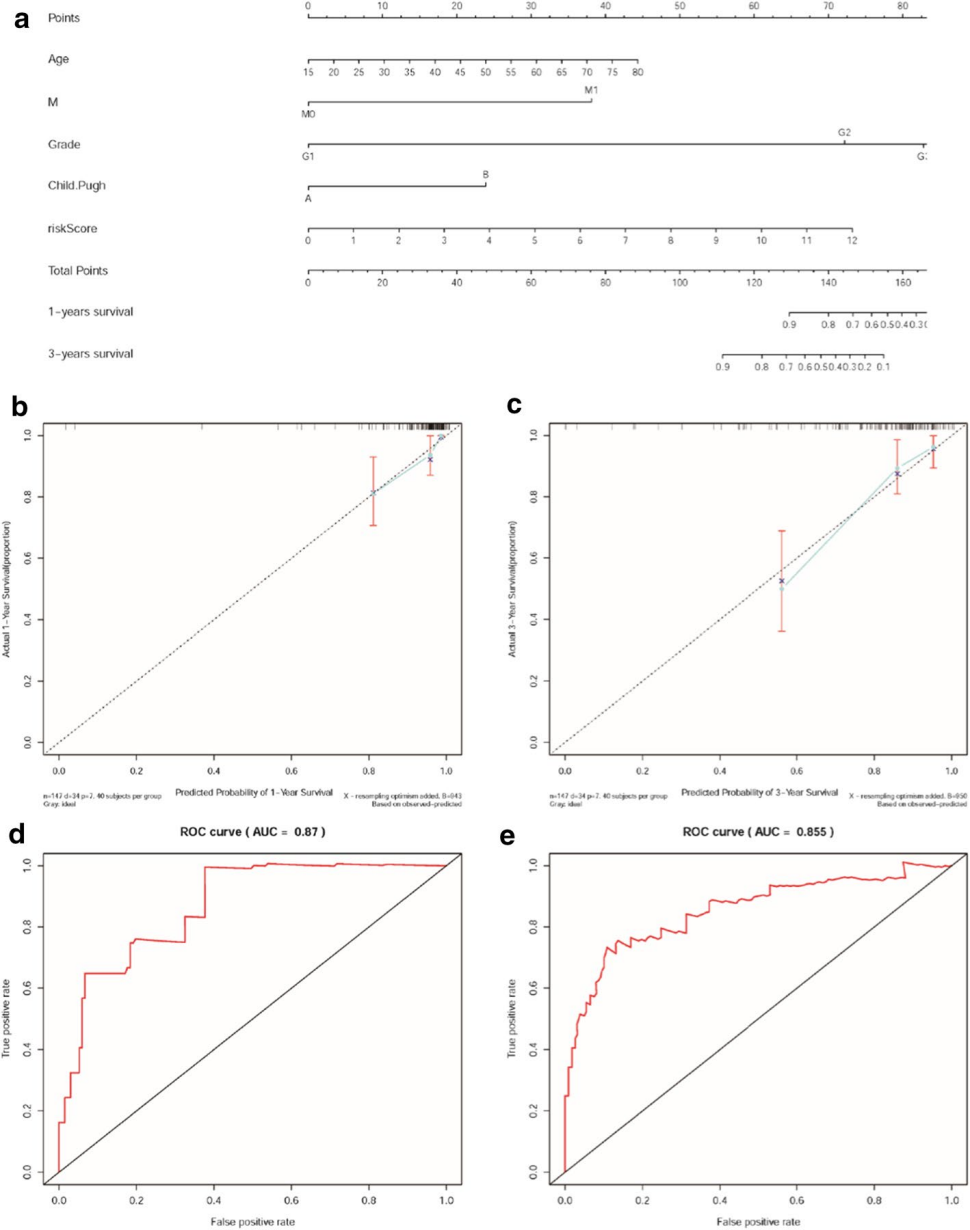
The performance evaluation and application of the nomogram. **a** Based on the total score which were calculated by summing the scores of each item to calculated the survival rate of HCC patients for 1- and 3-year. **b**, **c** The calibration curves of the nomogram in 1- and 3-year. **d**, **e** The ROC curves of nomogram in 1-and 3-year. The AUC of 1-and 3-year are 0.87 and 0.855 respectively

Based on previous multivariate Cox regression analysis, the risk score, M_stage, child_pugh, age, and Grade were both independent prognostic factors. Therefore, all of these factors were adopted into a nomogram which can assist in clinical interpretation of the constructed signature and be convenient to predict the survival rate of HCC patients. Based on the nomogram, the survival rate of 1- and 3-years can be assessed by summing the score of each item (Fig. 5a). The calibration curves of the nomogram indicates that the predicted survival rates of 1- and 3- years have superior accuracy (Fig. 5b, c). The area under the ROC curve (AUC) are 0.87, and 0.855 respectively (Fig. 5d, e).

### Functional analysis

**Fig. 6:**
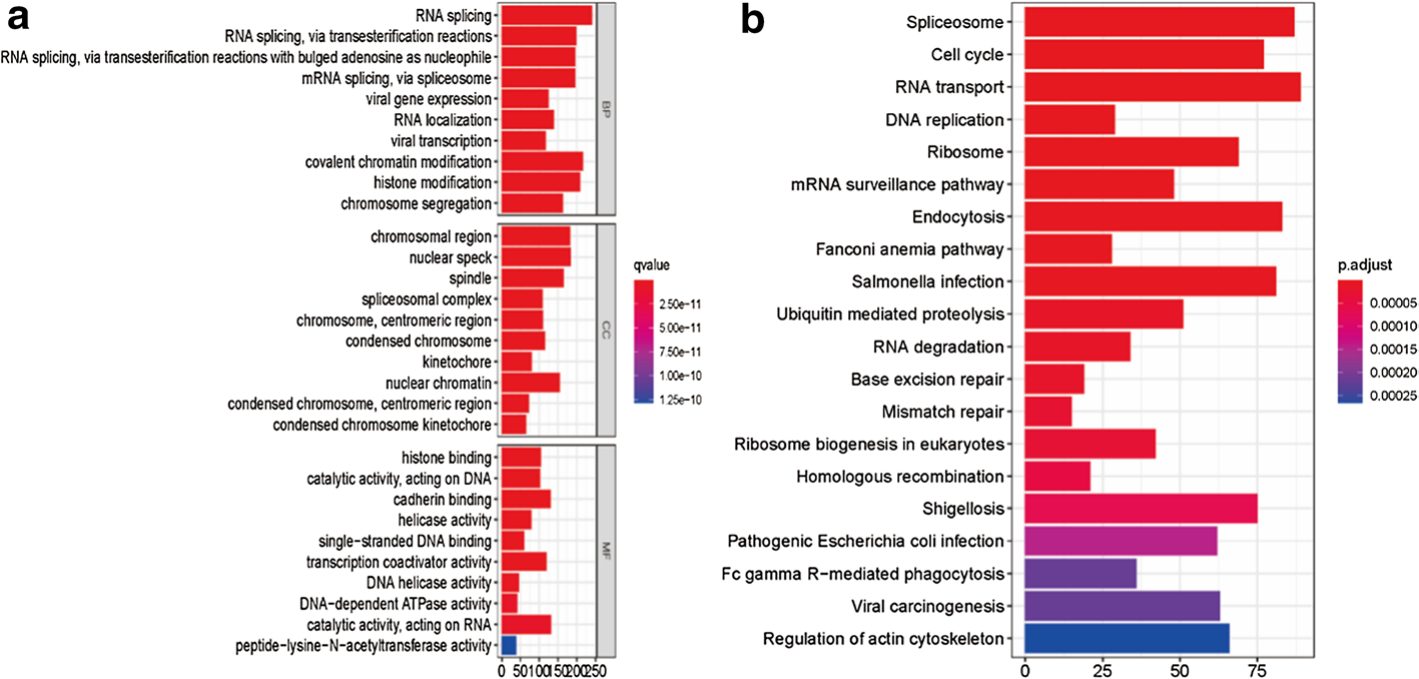
GO and KEGG pathway enrichment analyses. **a** Bar graph of GO enrichment analysis of the 7 autophagy-related lncRNA. **b** Bar graph of KEGG pathway enrichment analysis of the 7 autophagy-related lncRNAs. *GO* Gene Ontology, *KEGG* Kyoto Encyclopedia of Genes and Genomes

Under the inclusion criteria of |R| > 0.4 and *P* < 0.001, a total of 3580 genes that have a co-expression relationship with 7 prognostic autophagy-related lncRNAs were obtained (Additional file 3: Table S3). KEGG analysis shows that the 7 lncRNAs are directly or indirectly involved in Spliceosome, Cell cycle, RNA transport, DNA replication, Ribosome, mRNA surveillance pathway, and Endocytosis (Fig. 6b). GO results indicate that these lncRNAs may be related to the biological process of RNA splicing, mRNA splicing, RNA localization, covalent chromatin modification, and histone modification (Fig. 6a).

## Discussion

Autophagy, an evolutionary and conservative multistage lysosomal degradation process that promotes metabolism and healthy circulation, plays a complex and contradictory role in tumor formation and cancer treatment [11]. As a subclass of the ncRNAs family, lncRNAs play an indispensable role in various biological processes of tumorigenesis, which are considered a new type of biomarker for cancer diagnosis and prognosis widely concerned [44]. Although some studies have explored the impact of autophagy-related lncRNAs on the prognosis of HCC patients and constructed predictive signatures, the accuracy of each signature is different and the lncRNAs included are not all the same. Therefore, it is necessary to explore more autophagy-related lncRNAs that affect the prognosis of HCC patients to further discover specific regulatory networks.

In our study, autophagy-related lncRNAs were obtained by establishing the co-expression network of lncRNAs and autophagy genes. Univariate and multivariate Cox regression analyses were used to obtain the 7 prognostic autophagy-related lncRNAs, including PRRT3-AS1, RP11-479G22.8, RP11-73M18.8, LINC01138, CTD-2510F5.4, and RP11-324I22.4, and CTC-297N7.9. The analysis of the signature constructed by these 7 lncRNAs shows that this risk score has good survival prediction efficiency in HCC patients (*P* = 2.292e−10). Compared with traditional factors ALBI (AUC is 0.532), child_pugh (AUC is 0.573), Stage (AUC is 0.631) and AFP (AUC is 0.571), the forecasting advantage of risk score is significant (AUC is 0.786). On the contrary, the prognostic prediction value of the risk score in the ICC patient group is not obvious ehough (*P* = 4.301e−01, the AUC is 0.505). Moreover, the nomogram shows that the 1-year and 3-year survival rates of patients are obviously accuracy by the combined analysis of the risk score, child_pugh, age, M_stage, and Grade (The AUC of 1- and 3-years are 0.87, and 0.855). Therefore, the seven autophagy-related lncRNAs which constructed signature may become potential prognostic molecular markers and therapeutic targets for HCC patients.

On the one hand, as the only beneficial prognostic lncRNA in the prognostic prediction model, the gene alias of CTC-297N7.9 is lnc-TMEM220-1, which is an intergenic ncRNA. An HCC study showed that CTC-297N7.9 might be related to cofactor/chromatin/NAD binding and oxidoreductase/DNA-dependent ATPase activity [45]. Besides, due to the specific low expression and high methylation of TMEM220 in gastric cancer tissues [46], some scholars speculate that CTC-297N7.9 that located upstream of the protein-coding gene TMEM220, may be able to regulate the methylation of TMEM220 or participate in autophagy through its functional proteins, which in turn affects the prognosis of HCC patients [32]. In our research, we have speculated that the highly expressed CTC-297N7.9 may be an inhibitory factor in the progression of HCC. This speculation was confirmed in another study on liver cancer, and the high expression of CTC-297N7.9 often predicts better overall survival and disease-free survival [47]and indicates that CTC-297N7.9 may be one of the critical molecules to improve HCC patients’ survival, and it can be further explored in subsequent studies on HCC.

On the other hand, the 6 unfavorable prognostic lncRNAs in the prognostic prediction model have also been attached to various cancers. The official full name of PRRT3-AS1 is PRRT3 antisense RNA 1, as a non-protein-coding RNA, which is mainly expressed in liver tissue (RPKM 0.15), fat (RPKM 4.4), prostate (RPKM 3.3), and brain tissue (RPKM 3.0) [48]. In prostate cancer, Fan et al. confirmed that PRRT3-AS1 has a targeting relationship with PPARγ. Its silence can promote apoptosis autophagy and inhibit the proliferation, migration, and invasion of tumor cells through the mTOR signaling pathway [49]. Besides, PRRT3-AS1 is also considered to be related to GBM patients' prognosis [50]. RP11-479G22.8 is also known as lnc-ITGB1-1 in the LNCipedia database [51], and its transcription size is 2051 bp. Through the lncRNA disease prediction module of the lncRNASNP2 database [52], RP11-479G22.8 is closely related to HCC (*P* < 0.001). Therefore, RP11-479G22.8 is expected to be one of the potential indicators for prognostic prediction in HCC patients [45]. RP11-73M18.8 is a sense-intronic lncRNA with a transcript size of 811 bp, also known as lnc-ZFYVE21-3. Sense-intronic lncRNA is a sequence in the intron of the coding gene on the sense strand. It might harbor different histone modification at the transcription start site (TSS) than other ncRNAs [53], which indicated that these intronic lncRNAs maybe the novel biomarkers, such as type 2 diabetes mellitus [54]. LINC01138 is also a member of the sense intron ncRNA, located on chr1. Its abnormal expression has an important influence on the occurrence and development of several cancers. In prostate cancer (PCa), as a lncRNA that directly target AR, the high expression of LINC01138 can promote the proliferation of tumor cells and inhibit their apoptosis, which indicated that LINC01138 could be a diagnostic and prognostic marker for PCa [55] Besides, LINC01138 can increase the arginine methylation and protein stability of sterol regulatory element-binding protein one by interacting with PRMT5, thereby promoting lipid desaturation and cell proliferation in clear cell renal cell carcinoma and being associated with poor prognosis [56]. However. The knockdown of LINC01138 can inhibit the viability, proliferation, invasion, and migration and promotes apoptosis of gastric cancer cells through the LINC01138/miR-1273e/MAPK axis [57].In some studies related to HCC, high-expressed LINC01138 is not only significantly associated with poor survival [58] but also can interact with arginine methyltransferase 5 to promote cell proliferation, tumorigenicity, tumor invasion, and metastasis [59]. CTD-2510F5.4 is a 321 bp antisense lncRNA, also known as lnc-SKA2-1 in the LNCipedia database. Through the NPInter v4.0 database [60], we found that CTD-2510F5.4 mainly interacts with genes in the mRNA surveillance pathway and RNA transport pathway. The high expressed CTD-2510F5.4 also has a significant co-expression relationship with mRNAs of the cell cycle, DNA replication, and p53 signaling pathway [61]. It is closely related to the poor prognosis of patients with lung adenocarcinoma [62]. In gastric cancer, the highly expressed CTD-2510F5.4 may be an independent risk factor for tumors with pathological grade < III and no vascular or nerve infiltration [63]. RP11-324I22.4 is an antisense lncRNA; the gene alias is lnc-CUL2-3. As cancer or tumor suppressor genes, antisense lncRNAs play an essential role in the occurrence and development of human cancer [64–66]. Although there is currently no disease research related to RP11-324I22.4, antisense lncRNAs may certainly be the promising tumor biomarker and therapeutic target in future research.

Although the constructed signature shows superiority in predicting the prognosis of HCC patients compared with traditional indicators (ALBI, APF, child_pugh, and Stage) and the previously published signatures, there are still certain limitations. This study is only based on the TCGA database, and there are no suitable datasets in GEO (Gene Expression Omnibus, https://www.ncbi.nlm.nih.gov/geo/) [67] and ICGC (International cancer genome consortium, https://dcc.icgc.org/) [68] databases to verify the risk prediction signature. Furthermore, the research is only conducted at the level of bioinformatics; a comprehensive in vitro experiment is needed further to explore the regulatory mechanism of these autophagy lncRNAs.

## Conclusions

In general, the constructed risk prediction model of autophagy-related lncRNAs (PRRT3-AS1, RP11-479G22.8, RP11-73M18.8, LINC01138, CTD-2510F5.4, and RP11-324I22.4, and CTC-297N7.9) based on autophagy genes is robust and promising. The lncRNAs in this model can be used as potential biomarkers for the prognosis of HCC, which will help the individualized treatment of HCC.

## Supplementary Information

**Additional file 1: Table S1.** The co-expression result of autophagy-related lncRNAs and autophagy genes.

**Additional file 2: Table S2.** The 39 strong prognostic autophagy-related lncRNAs were identified by univariate Cox regression analysis and product-limit method.

**Additional file 3: Table S3.** The 3580 genes have co-expression with 7 prognostic autophagy-related lncRNAs.

## Data Availability

The entire sequencing profile data and the clinical data of HCC and ICC patients in this study come from The Cancer Genome Atlas (TCGA, https://cancergenome.nih.gov/) database, and the autophagy-related genes were obtained from Human Autophagy Database (HADb, http://www.autophagy.lu/).
